# Clinical Feature of Men Who Benefit from Dose Escalation of Naftopidil for Lower Urinary Tract Symptoms: A Prospective Study

**DOI:** 10.1155/2011/804583

**Published:** 2011-04-05

**Authors:** Takaki Mizusawa, Noboru Hara, Kenji Obara, Etsuko Isahaya, Yuki Nakagawa, Kota Takahashi

**Affiliations:** ^1^Division of Urology, Department of Regenerative and Transplant Medicine, Graduate School of Medical and Dental Sciences, Niigata University, Asahimachi 1, Niigata 951-8510, Japan; ^2^Department of Urology, Kido Hospital, Niigata, Kamikido 5, Niigata 950-0891, Japan

## Abstract

*Objectives*. To examine the feature of men who benefit from dose escalation of naftopidil for lower urinary tract symptoms (LUTSs). *Methods*. Based on the IPSS, men reporting LUTS were prospectively studied using 50 mg/day of naftopidil for the first 4 weeks; satisfied patients continued its 50 mg/day (*n* = 11), and those reporting unsatisfactory improvement received its 75 mg/day (*n* = 35) for the next 4 weeks. *Results*. The 75 mg group showed improvement in the total IPSS and QOL score in a dose-dependent manner (at 4 weeks: *P* < .001, at 4 weeks versus 8 weeks: *P* < .05). In the 50 mg group, both scores reduced at 4 weeks, thereafter unchanged. The baseline slow stream score alone was higher in the 75 mg group (*P* = .013). The rate of change in the QOL score during the initial 4 weeks (ΔQOL) and Δnocturia was smaller in the 75 mg group (*P* < .05). *Conclusions*. Men with high slow stream score and unsatisfactory improvement in nocturia may benefit from dose escalation of naftopidil.

## 1. Introduction

Lower urinary tract symptoms (LUTSs) impair the health-related quality of life (QOL) [1–4]. The prevalence and severity of LUTS increase with age, and many middle-aged to elderly people show various levels of LUTS [4, 5]. Voiding symptoms are more popular in men than in women [6, 7], and, correspondingly, the causative condition associated with male LUTS is represented by benign prostatic hyperplasia (BPH). The treatment theory for BPH is thus targeting voiding symptoms mainly based on two types of lower urinary tract obstruction: mechanical urinary tract obstruction by the enlarged prostate and functional constriction of the urethral and prostatic smooth muscle via sympathetic *α*_1_-stimulants/*α*_1_-adrenoreceptors interaction. Accordingly, *α*_1_-adrenoreceptor antagonists are widely applied as the first-line in current therapeutic practice for BPH. On the other hand, it is also important to manage not only voiding symptoms but also storage symptoms in men with LUTS. Alpha_1_-adrenoceptor antagonists have also been known to alleviate storage symptoms, although the mechanism of storage symptoms as well as how mentioned agents are involved in improvement of them has not been fully elucidated [3, 4, 7, 8].

Naftopidil has a high affinity for *α*_1D_-adrenoceptor; *α*_1_-adrenoceptor antagonists with high selectivity for *α*_1A_-adrenoceptors has been thought to be more effective in treatment of LUTS [8, 9], whereas recent basic and clinical studies also showed the therapeutic potential of *α*_1_-adrenoceptor antagonists with high selectivity for *α*_1D_-adrenoceptors [10]. Indeed, naftopidil has been applied for men having BPH-associated LUTS and has been regarded as a second-generation *α*_1_-adrenoceptor antagonist [11]. While *α*_1A_-adrenoceptors play a critical role in relaxing smooth muscle of the prostate and urethra and improve obstructive/voiding symptoms [8, 9], storage and bladder irritability symptoms are predominantly *α*_1D_-adrenoceptor-associated conditions [10]. Although unfavorable/adverse effects by naftopidil on blood pressure/cardiovascular system are infrequent, 50 mg/day of peroral naftopidil is the initial treatment setting in Asian populations generally [11]. A recent case-control study reported that the setting of 75 mg/day showed a higher therapeutic efficacy compared with that of 25 or 50 mg/day of naftopidil [11, 12]. Yet, there have been few studies that examined the treatment efficacy and safety of 75 mg/day of peroral naftopidil in longitudinal comparison with those of 50 mg/day; such approach may possibly characterize patients who benefit from dose escalation of naftopidil without compromising safety in clinical practice. Thereby, key/leading symptoms or factors impairing LUTS-related QOL may be underscored. In the present study, we prospectively studied men having moderate-to-severe LUTS in a longitudinal approach with dose escalation from 50 mg to 75 mg of naftopidil once a day to examine the feature of patients who prefer and benefit from dose escalation of naftopidil, and to identify which symptom and improvement thereof reflect the LUTS-related QOL.

## 2. Methods

### 2.1. Patients

In total, 53 patients who presented at Niigata University Hospital and associated institutions with the International Prostate Symptom Score (IPSS) of 8 or higher were prospectively enrolled between September 2007 and July 2009. The performance status was good (World Health Organization Performance Status Score zero) in all of them. The study had a longitudinal design, and the procedure for this research project was approved by the Ethics Committee of our institution. Informed consent was obtained from all of the patients. Exclusion criteria were patients with a history of bladder cancer, prostate cancer, or pelvic irradiation, along with those diagnosed with bladder stone, untreated definite urinary tract infections, and neurogenic bladder. Patients who had already been treated for lower urinary tract dysfunctions with surgery or any intervention were also excluded.

### 2.2. Examinations and Questionnaires

At the initial visit, the patients filled in the baseline International Prostate Symptom Score (IPSS) sheets handed to them when visiting the outpatient clinic. General blood and biochemical examinations, urinalysis, residual urine volume measurement using an ultrasonographic instrument, and ultrasonographic examinations of the upper and lower urinary tracts were performed in all patients. Those with microscopic hematuria received a urinary cytological examination; all of them showed a normal smear.

### 2.3. Dose Escalation of Naftopidil and Longitudinal Assessment of IPSS

All patients were treated with 50 mg of naftopidil once a day for the first 4 weeks and reported the IPSS after administration. Patients without adverse events who reported unsatisfactory improvement of LUTS and preferred advanced treatment based on a question “are you satisfied with your current urinary state, yes or no?” received 75 mg of naftopidil once a day for the next 4 weeks (75 mg group) and reported the IPSS again following treatment (8 weeks after starting treatment). Those having satisfactory improvement with the initial treatment at 50 mg/day continued 50 mg of naftopidil (50 mg group) and also reported IPSS in the next 4 weeks (8 weeks after starting treatment). Adverse events were assessed at every visit.

### 2.4. Statistical Analysis

In addition to the chi-square test and pared *t*-test, the Welch-corrected *t*-test was used to compare unpaired parameters between two subgroups, and Tukey's honestly significant difference (HSD) was used for the comparison of values among 3 or more subgroups. Correlations between parameters were analyzed using Spearman's rank correlation coefficient analysis (rs). Multilinear logistic regression model, which produces prediction formula with a smaller margin of error, was used for the identification of independent significant correlations among continuous or stepwise variables. The test was two-sided, and *P* < .05 was considered significant. All analyses were performed using SPSS version 11.0J (SPSS Inc., Chicago, IL, USA) in a Windows-based computer.

## 3. Results

### 3.1. Adverse Events and Patients' Demographics

Data were available in 53 patients, and, of these, treatment was discontinued due to adverse effects at the dose of 50 mg/day and 75 mg/day in 5 and 2 patients (9.4% and 5.4%), respectively, at 50 mg: dizziness in 2, slight stagger in 1, orthostatic hypotension in 1, and orthostatic syncope in 1, and the events soon disappeared by drug discontinuation; at 75 mg, 2 patients reported slight stagger, and these disappeared soon after dose reduction to 50 mg. Excluding these subjects, 46 patients comprised the final study groups, and 35 and 11 men were included in the 75 mg and 50 mg groups, respectively. Demographics and the baseline IPSS of the patients were shown in Table 1. Patients' age, prostate volume, residual urine volume, total IPSS, and storage, voiding, and quality-of-life (QOL) scores were equally distributed between the 75 mg and 50 mg groups (Table 1). For each domain of the baseline IPSS, the score for slow stream alone was higher in the 75 mg group than in the 50 mg group (3.6 ± 1.6 versus 2.1 ± 1.9, *P* = .013).

### 3.2. Alteration of the IPSS and QOL Score during the Observation Period

**Figure 1:**
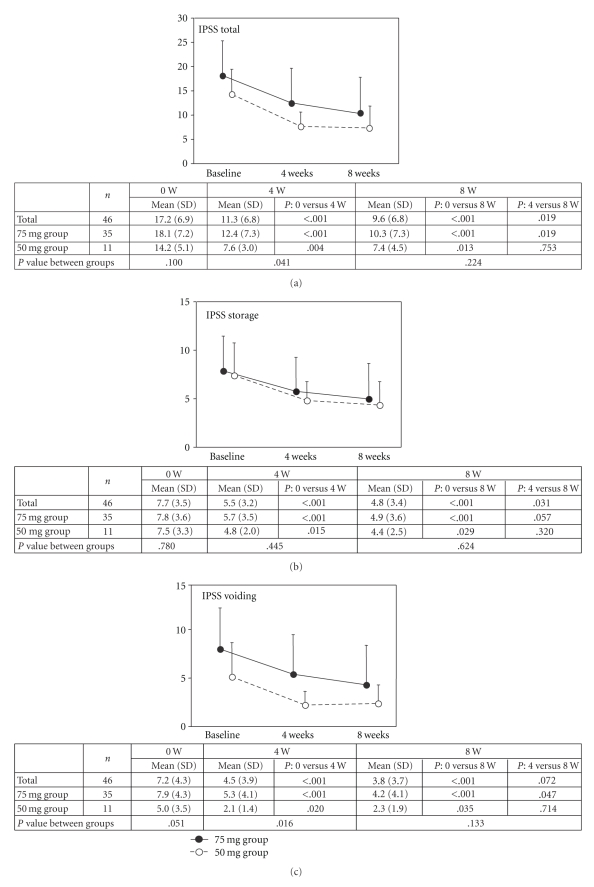

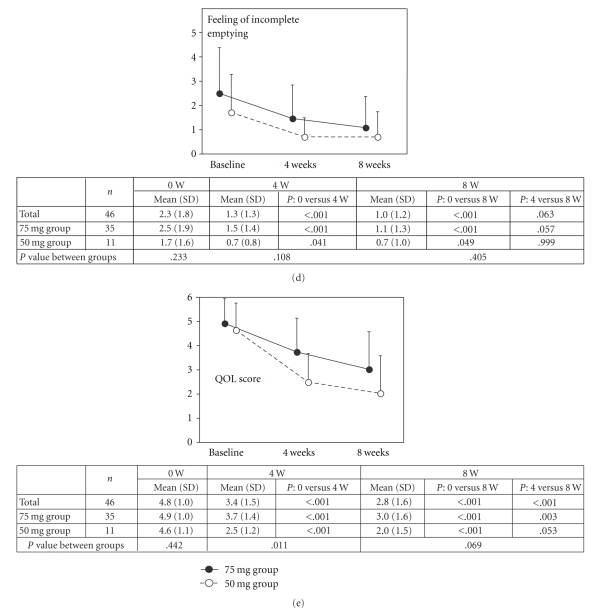
Alteration of the total IPSS (a), storage (b), voiding (c), postmicturition (domain for feeling of incomplete emptying) (d), and QOL (e) scores in men with dose escalation from 50 mg/day to 70 mg/day of naftopidil (75 mg group) and those maintained with its 50 mg/day (50 mg group).

The alteration of the total IPSS, storage score, voiding score, postmicturition score (domain for feeling of incomplete emptying), and QOL score was presented in Figures 1(a)–1(e). The 75 mg group showed improvement in all of these in a time-dependent and dose-dependent manner (baseline versus 4 weeks after treatment: *P* < .001 in all; 4 weeks after treatment versus 8 weeks after treatment: *P* = .019, *P* = .057, *P* = .047, *P* = .057, and *P* = .003, resp.), although the difference in the storage and postmicturition scores were of borderline significance between at 4 weeks and 8 weeks (Figures 1(b) and 1(d)). In the 50 mg group, all of these scores reduced 4 weeks after treatment compared with those at baseline (*P* = .004, *P* = .015, *P* = .020, *P* = .041, and *P* < .001, resp.); these scores were not different between 4 weeks and 8 weeks after treatment (Figures 1(a)–1(e)), although the difference was of borderline significance regarding the QOL score (*P* = .053).

### 3.3. Analyses Concerning the Feature of the 75 mg Group

**Figure 2:**
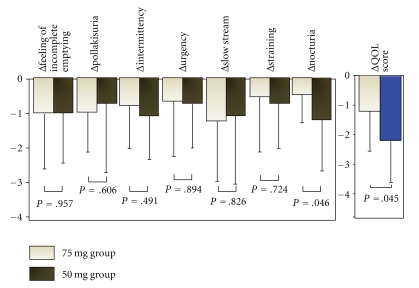
The rate of change in the QOL score (ΔQOL) and each domain of the IPSS (Δ each domain) with 50 mg/day of naftopidil during the first 4 weeks.

We further studied the clinical feature of men in the 75 mg group in comparison with the 50 mg group to identify those who benefit from 75 mg treatment. We hypothesized that the low degree of satisfaction concerning some specific symptoms with 50 mg/day of naftopidil might be associated with the motive for patients to select the dose escalation. We first verified that the rate of change in the QOL score during the initial 4 weeks (ΔQOL) in the 75 mg group was smaller than that in the 50 mg group (*P* = .045, Figure 2, right side columns). We subsequently analyzed the rate of change in each domain of the IPSS during the initial 4 weeks to examine which Δ domain potentially reflected ΔQOL, and found that the rate of change in nocturia (Δnocturia) alone was smaller in the 75 mg group than in the 50 mg group (*P* = .046, Figure 2). With Spearman's rank correlation coefficient analysis, the baseline score for nocturia was correlated with the QOL score in the overall patients (rs. = 0.459, *P* = .002) and in the 75 mg group (rs. = 0.462, *P* = .007), but the relationship was not significant in the 50 mg group despite the high correlation coefficient (rs. = 0.482, *P* = .127). The score for nocturia 4 weeks after treatment was also correlated with the QOL score in the overall patients (rs. = 0.459, *P* = .002) and in the 75 mg group (rs. = 0.475, *P* = .006); the relationship was not significant in the 50 mg group with the high correlation coefficient (rs. = 0.508, *P* = .108). Δnocturia was correlated with ΔQOL in the overall patients (rs. = 0.562, *P* < .001) and both in the 75 mg and 50 mg groups (rs. = 0.437, *P* = .011 and rs. = 0.663, *P* = .036, resp.). With multivariate analysis employing multilinear logistic regression model, Δnocturia alone was an independent value correlated with ΔQOL in all 46 patients (*r* = 0.542, *P* = .006). In each group, however, this model failed to identify significant independent correlations (data not shown).

## 4. Discussion

Funahashi and colleagues reported the efficacy of 75 mg/day of naftopidil in patients with BPH who did not show improvement with 50 mg/day of naftopidil [12]. In their study, 40 of the 122 patients received 75 mg/day of naftopidil; prostate volume of the 9 responders was larger than that of the 31 nonresponders, and the authors concluded possible advantage of the dose escalation to 75 mg/day of naftopidil in patients with BPH. The present study design was similar to that of Funahashi et al. [12], but, in our study, a higher fraction of patients (76%) experienced dose escalation based on the satisfaction in improvement of LUTS. We showed that the total IPSS, storage, voiding, postmicturition, and QOL scores in the 75 mg group decreased in a time-dependent and dose-dependent manner, although the difference in the postmicturition and storage scores was of borderline significance between at 4 weeks and 8 weeks (Figure 1(a)–1(e)). In the 50 mg group, these scores were similar between 4 weeks and 8 weeks after treatment, possibly suggesting dose-dependent rather than time-dependent effects of naftopidil in the 75 mg group; the number of patients enrolled in the 50 mg group was small to draw a definite conclusion. Additionally, adverse effects did not differ between the two settings, supporting the safety of 75 mg/day of this agent.

The present results showed that patients selecting 75 mg therapy had higher scores for the slow stream domain at baseline (mean 3.6, Table 1), and those with a baseline score of 3 or higher for slow stream are probably feasible candidates who benefit from dose escalation of naftopidil. Additionally, the 75 mg group seemingly had a higher IPSS voiding score at baseline and larger prostate volume than the 50 mg group; the small number of patients in the 50 mg group might lead to the bias. Thus, it is suggested that severer bladder outlet resistance was associated with unsatisfactory improvement of symptoms during 50 mg treatment in the 75 mg group. Naftopidil also has an *α*_1A_-adrenogenic activity, although it is weaker than that of other *α*_1_-adrenoreceptor antagonists such as tamsulosin. Naftopidil is *α*_1_-adrenoreceptor selective in the order of *α*_1D _> *α*_1A _≥ *α*_1B_, and it has about 3-fold selectivity for *α*_1D_-adrenoreceptors compared with that for *α*_1A_-adrenoreceptors [8], suggesting that dose escalation of naftopidil possibly could evoke more *α*_1A_-adrenoreceptor reactions in relaxing the prostate/urethra smooth muscle and alleviating voiding symptoms. For men with bladder outlet obstruction or reporting moderate-to-severe voiding symptoms, the initial appropriate dose of naftopidil may be 75 mg/day. 

Our study also suggested the dose-dependent efficacy of naftopidil for storage symptoms in the 75 mg groups (Figure 1(b)), although the difference was of borderline significance between 4 weeks and 8 weeks after treatment. Interestingly, analyses for the degree of satisfaction showed that ΔQOL during the first 4 weeks with 50 mg/day of naftopidil in the 75 mg group was smaller than that in the 50 mg group (*P* = .045, Figure 2) and that Δnocturia during the first 4 weeks may possibly be an important parameter for the degree of satisfaction (*P* = .046, Figure 2). We moreover verified the correlation between the QOL and nocturia scores at baseline or 4 weeks after 50 mg treatment with Spearman's rank correlation coefficient analysis in the overall patients and 75 mg group, but it was not significant in the 50 mg group. We could not draw a definite conclusion concerning this result in the 50 mg group due to the small number of patients, however; Δnocturia was correlated with ΔQOL both in the 75 mg and 50 mg groups (rs. = 0.437, *P* = .011 and rs. = 0.663, *P* = .036, resp.). These results also suggest the possible importance of improvement in nocturia for improvement in QOL by naftopidil. Effectiveness of naftopidil at a high dose for voiding symptoms can be explained by pharmacokinetics/pharmacodynamics of the agent in vivo and its effect on *α*_1_-adrenoceptor antagonists/*α*_1_-adrenoreceptors interaction [13]. Storage symptoms are considered to represent those of BPH-related overactive bladder [14], and most recent basic studies on *α*_1_-adrenoceptor antagonists/*α*_1_-adrenoreceptors in the lower urinary tract may account for the dose-dependent efficacy of naftopidil for improving storage symptoms [14, 15]. Unfortunately, our study cannot clarify the reason for improved storage symptoms including nocturia with naftopidil; the complexity of the mechanism of nocturia made us refrain from explicating this phenomenon [16]. The smaller voiding score and prostate volume in the 50 mg group suggests another possible difference in the etiology of LUTS between the two groups; LUTS in the 50 mg group may be associated with bladder-related conditions. As described elsewhere, naftopidil is an agent with relatively high selectivity for *α*_1D_-adrenoreceptors [17]. It has been reported that not only *α*_1A_- but also *α*_1D_-adrenoreceptor antagonists effectively treat male LUTS and BPH-related symptoms, but the *α*_1D_-relevant mechanism has not been fully elucidated. Urinary-function-related *α*_1D_-adrenoreceptors are distributed in the prostate, bladder, and spinal cord [17]. Alpha_1_-adrenoreceptors most frequently expressed in the human bladder are the *α*_1D_-type, which has recently been regarded as an important subtype associated with LUTS [17]. In a bladder outlet obstruction model using rats, the increase in bladder detrusor muscle was associated with the higher expression of *α*_1D_-adrenoreceptors both in mRNA and protein levels [18]. In another study on the central nervous system, intrathecally injected naftopidil reduced the intensity of bladder constriction in rats, suggesting the relevance of *α*_1D_-adrenoreceptors in the spinal cord center [19]. With frequency/volume analysis and filling cystometry in *α*_1D_ knockout mice, Chen and colleagues reported that *α*_1D_-adrenoreceptor subtype plays a critical role in regulating bladder function, theoretically supporting clinical observations such as effectiveness of naftopidil for treating storage symptoms [20]. Further clinical and basic approaches are thus warranted to elucidate the various effects of this agent.

The present study had several limitations. The small number of participants in the current study led to a limited capacity. Randomized double-blind studies in a large volume could have provided higher data quality supported by a higher evidence level. Also, studies on pharmacokinetics/pharmacodynamics were not performed in our study.

In conclusion, the present study with dose escalation from 50 mg/day to 75 mg/day of naftopidil showed that 75 mg/day of naftopidil was useful for alleviating storage, voiding, and postmicturition symptoms in men without compromising safety. The high score for slow stream at baseline and unsatisfactory improvement in nocturia with 50 mg/day of naftopidil may be important factors for men with LUTS, who prefer and benefit from dose escalation of this agent. Further studies are warranted to elucidate the various effects of naftopidil for male LUTS.

##  Conflict of Interests 

The authors declare no conflict of interests. This work has not been funded by any commercial company or organization. 

## Figures and Tables

**Table 1:** Patients' demographics and the baseline International Prostate Symptom Score (IPSS).

	Total	75 mg group	50 mg group	*P* value
	(*n* = 46)	(*n* = 35)	(*n* = 11)	(75 mg versus 50 mg)
Age, mean (years)	71.7 ± 9.4	72.1 ± 9.2	70.2 ± 10.5	.559
Prostate volume, mean (mL)	31.7 ± 24.8	34.6 ± 24.9	25.0 ± 24.8	.403
Residual urine, mean (mL)	24.0 ± 41.5	24.5 ± 43.3	22.4 ± 36.1	.899
IPSS total	17.2 ± 6.9	18.1 ± 7.2	14.2 ± 5.1	.100
Storage score	7.7 ± 3.5	7.8 ± 3.6	7.5 ± 3.3	.780
Pollakisuria	3.0 ± 1.6	3.1 ± 1.6	2.8 ± 1.8	.602
Urgency	1.9 ± 1.8	2.0 ± 1.9	1.5 ± 1.4	.469
Nocturia	2.8 ± 1.3	2.7 ± 1.3	3.1 ± 1.4	.385
Voiding score	7.2 ± 4.3	7.9 ± 4.3	5.0 ± 3.5	.051
Intermittency	2.2 ± 1.9	2.4 ± 2.0	1.7 ± 1.7	.334
Slow stream	3.2 ± 1.8	3.6 ± 1.6	2.1 ± 1.9	.013
Straining	1.7 ± 1.8	1.9 ± 1.9	1.2 ± 1.3	.242
Postmicturition score*	2.3 ± 1.8	2.5 ± 1.9	1.7 ± 1.6	.233
QOL score	4.8 ± 1.0	4.9 ± 1.0	4.6 ± 1.1	.442

Score: mean ± standard deviation (SD); postmicturition score*, domain for feeling of incomplete emptying.

## Abbreviations

LUTS: Lower urinary tract symptoms
BPH: Benign prostatic hyperplasia
IPSS: International Prostate Symptom Score.
